# Comparison of Racial, Ethnic, and Geographic Location Diversity of Participants Enrolled in Clinic-Based vs 2 Remote COVID-19 Clinical Trials

**DOI:** 10.1001/jamanetworkopen.2021.48325

**Published:** 2022-02-14

**Authors:** Jenell Stewart, Meighan L. Krows, Torin T. Schaafsma, Kate B. Heller, Elizabeth R. Brown, Jim Boonyaratanakornit, Clare E. Brown, Hannah Leingang, Caroline Liou, Anna Bershteyn, Mark D. Schwartz, Vaidehi Agrawal, DeAnna Friedman-Klabanoff, Stephen Eustace, Helen C. Stankiewicz Karita, Michael K. Paasche-Orlow, Patricia Kissinger, Sybil G. Hosek, Helen Y. Chu, Connie Celum, Jared M. Baeten, Anna Wald, Christine Johnston, Ruane V. Barnabas

**Affiliations:** 1 Department of Global Health, University of Washington, Seattle; 2 Department of Medicine, University of Washington, Seattle; 3 Department of Biostatistics, University of Washington, Seattle; 4 Department of Vaccine and Infectious Disease Division, Fred Hutchinson Cancer Research Center, University of Washington, Seattle; 5 Department of Population Health, New York University Grossman School of Medicine, New York, New York; 6 Center for Vaccine Development and Global Health, University of Maryland School of Medicine, Baltimore; 7 PRA Health Sciences, Digital Health Engagement, Raleigh, North Carolina; 8 Department of Medicine, Boston University School of Medicine, Boston Medical Center, Boston, Massachusetts; 9 Department of Epidemiology, Tulane School of Public Health and Tropical Medicine, New Orleans, Louisiana; 10 Department of Psychiatry, John H. Stroger Jr Hospital of Cook County, Chicago, Illinois; 11 Department of Epidemiology, University of Washington, Seattle; 12 HIV Clinical Development at Gilead Sciences, Foster City, California; 13 Department of Laboratory Medicine, University of Washington, Seattle; 14 Department of Pathology, University of Washington, Seattle

## Abstract

**Importance:**

Racial and ethnic diversity among study participants is associated with improved generalizability of clinical trial results and may address inequities in evidence that informs public health strategies. Novel strategies are needed for equitable access and recruitment of diverse clinical trial populations.

**Objective:**

To investigate demographic and geographical location data for participants in 2 remote COVID-19 clinical trials with online recruitment and compare with those of a contemporaneous clinic-based COVID-19 study.

**Design, Setting, and Participants:**

This cohort study was conducted using data from 3 completed, prospective randomized clinical trials conducted at the same time: 2 remotely conducted studies (the Early Treatment Study and Hydroxychloroquine COVID-19 Postexposure Prophylaxis [PEP] Study) and 1 clinic-based study of convalescent plasma (the Expanded Access to Convalescent Plasma for the Treatment of Patients With COVID-19 study). Data were collected from March to August 2020 with 1 to 28 days of participant follow-up. All studies had clinical sites in Seattle, Washington; the 2 remote trials also had collaborating sites in New York, New York; Syracuse, New York; Baltimore, Maryland; Boston, Massachusetts; Chicago, Illinois; New Orleans, Louisiana; and Los Angeles, California. Two remote trials with inclusive social media strategies enrolled 929 participants with recent SARS-CoV-2 exposure (Hydroxychloroquine COVID-19 PEP Trial) and 231 participants with COVID-19 infection (Early Treatment Study); the clinic-based Expanded Access to Convalescent Plasma for the Treatment of Patients With COVID-19 study enrolled 250 participants with recent COVID-19 infection. Data were analyzed from April to August 2021.

**Interventions:**

Remote trials used inclusive social media strategies and clinician referral for recruitment and telehealth, courier deliveries, and self-collected nasal swabs for remotely conducted study visits. For the clinic-based study, participants were recruited via clinician referral and attended in-person visits.

**Main Outcomes and Measures:**

Google Analytics data were used to measure online participant engagement and recruitment. Participant demographics and geographical location data from remote trials were pooled and compared with those of the clinic-based study. Statistical comparison of demographic data was limited to participants with COVID infections (ie, those in the remotely conducted Early Treatment Study vs those in the clinic-based study) to improve accuracy of comparison given that the Hydroxychloroquine COVID-19 PEP Trial enrolled participants with COVID-19 exposures and thus had different enrollment criteria.

**Results:**

A total of 1410 participants were included. Among 1160 participants in remote trials and 250 participants in the clinic-based trial, the mean (range) age of participants was 39 (18-80) years vs 50 (19-79) years and 676 individuals (58.3%) vs 131 individuals (52.4%) reported female sex. The Early Treatment Study with inclusive social media strategies enrolled 231 participants in 41 US states with increased rates of racial, ethnic, and geographic diversity compared with participants in the clinic-based study. Among 228 participants in the remotely conducted Early Treatment Study with race data vs participants in the clinic-based study, 39 individuals (17.1%) vs 1 individual (0.4%) identified as Alaska Native or American Indian, 11 individuals (4.8%) vs 22 individuals (8.8%) identified as Asian, 26 individuals (11.4%) vs 4 individuals (1.6%) identified as Black, 3 individuals (1.3%) vs 1 individual identified as Native Hawaiian or Pacific Islander, 117 individuals (51.3%) vs 214 individuals (85.6%) identified as White, and 32 individuals (14.0%) vs 8 individuals (3.2%) identified as other race (*P* < .001). Among 230 individuals in the Early Treatment Study vs 236 individuals in the clinic-based trial with ethnicity data, 71 individuals (30.9%) vs 11 individuals (4.7%) identified as Hispanic or Latinx (P<.001). There were 29 individuals in the Early Treatment Study with nonurban residences (ie, rural, small town, or peri-urban; 12.6%) vs 6 of 248 individuals in the clinic-based trial with residence data (2.4%) (*P* < .001). In remote trial online recruitment, the highest engagement was with advertisements on social media platforms; among 125 147 unique users with age demographics who clicked on online recruitment advertisements, 84 188 individuals (67.3%) engaged via Facebook.

**Conclusions and Relevance:**

These findings suggest that remote clinical trials with online advertising may be considered as a strategy to improve diversity among clinical trial participants.

## Key Points

**Question:**

Were racial, ethnic, and geographic location demographics of participants enrolled in 2 remote clinical trials with online recruitment more diverse compared with those of participants in a clinic-based COVID-19 study?

**Findings:**

This cohort study was a secondary analysis of 1410 participants enrolled in 3 COVID-19 studies conducted in 2020 during the early COVID-19 pandemic and found that remote clinical trials with online recruitment had increased racial, ethnic, and geographic location diversity among study participants.

**Meaning:**

These findings suggest that remotely conducted trials with inclusive social media recruitment may be considered as potential components to address insufficient representation and enrollment of diverse populations in clinical trials while continuing to research this important aspect of clinical trials.

## Teaser

This cohort study compares racial, ethnic, and geographic location diversity of participants in a clinic-based vs 2 remote COVID-19 clinical trials.

## Introduction

Enrollment of diverse populations in clinical trials is associated with more equitable opportunities and improved generalizability of results.^1,2^ Mistrust engendered by a long-standing track record of societal mistreatment experienced by Black, Hispanic, and Indigenous study participants is associated with underrepresentation of these groups in clinical trials.^3^ Issues such as proximity to trial sites, access to transportation, and time flexibility needed to participate are also associated with these disparities.^2,4,5^ Remote trials may be associated with reductions in some of these barriers.^6^

The COVID-19 pandemic highlights preexisting structural racism and disparities, with the burden of COVID-19 morbidity and mortality disproportionately impacting people of color.^7,8^ In 2020, US COVID-19 mortality for Hispanic people aged younger than 65 years was 7-fold to 9-fold higher than that among non-Hispanic White people aged younger than 65 years.^9^ Furthermore, access to investigational COVID-19 agents are largely restricted to trial participants enrolled at a limited number of sites despite widespread transmission of COVID-19.^5^ We evaluated the racial, ethnic, and geographic diversity of participants in 2 remote randomized clinical trials with online recruitment^10,11^ and compared to that of a clinic-based COVID-19 randomized clinical trial in the same period.^12^

## Methods

This cohort study is reported following the Strengthening the Reporting of Observational Studies in Epidemiology (STROBE) reporting guideline. This research was conducted with approval from the Western Institutional Review Board with reliance agreements with collaborating institutions, and all participants gave written informed consent. We conducted a cohort study using 2 remotely conducted randomized clinical trials for SARS-CoV-2 prevention and treatment (the Early Treatment Study [NCT04354428]^10^ and Hydroxychloroquine COVID-19 Postexposure Prophylaxis [PEP] Study [NCT04328961]^11^) and 1 clinic-based randomized clinical trial (the Expanded Access to Convalescent Plasma for the Treatment of Patients With COVID-19 study [NCT04338360]).^12^ For remote studies, between March and August 2020, participants were recruited through national online advertising (ie, Facebook, Google, Twitter, and Reddit), clinician referral, and self-referral through the study website. We tracked the COVID-19 positivity rate by zip code and focused recruitment to areas with rapidly increasing incidence rates.^13^ This dynamic digital media strategy and advertising outreach was tailored weekly. Advertising featured people representative of populations disproportionally impacted by the COVID-19 pandemic. We tracked engagement using Google Analytics web analytics service (Google). The clinic-based trial recruited through clinician referral alone. Inclusion criteria for remote Early Treatment Study and clinic-based Expanded Access to Convalescent Plasma for the Treatment of Patients With COVID-19 study participants included adults with confirmed COVID-19 infection.

Remote trials were conducted with a completely remote study design using Health Insurance Portability and Accountability Act of 1996 (HIPAA)–compliant telemedicine, electronic informed consent, and electronic data capture. Consent was offered in English and Spanish, and study staff fluent in Spanish were available. Couriers delivered study kits to participants containing study medication and supplies (eg, daily nasal swabs) as previously described.^10,14^ Participants received instructions in print and from email reminders, prerecorded videos, and demonstrations via telemedicine. Participants entered medication use and symptom data in an electronic study database (Research Electronic Data Capture [REDCap]), with real-time operational support for technical difficulties, missed doses of study medication, late daily surveys, and shipment forecasting.

We collected demographic, including race and ethnicity data (self-reported in all trials), and geographic data by self-report using electronic surveys. Community classification was assigned according to US Department of Agriculture rural-urban commuting area codes, which we classified as urban, peri-urban, small town, or rural by participant zip code.^15^ Survey options for the race category included Alaska Native or American Indian, Asian, Black or African American, Native Hawaiian or Pacific Islander, White, and other if racial identity was not captured in available options. Survey options for ethnicity included yes or no to Hispanic or Latinx. Outcomes for White individuals were compared with those of members of other racial and ethnic groups because this study was conducted specifically to address the overrepresentation of people who identify as White in trials. We compiled a map of participant locations using 3-digit ZIP Code Tabulation Areas.

Descriptive data were analyzed by *t* test and Fisher exact test with a significance level of *P* < .05 by 2-sided tests. Statistical comparison of demographic data was limited to participants with COVID infections (ie, those in the remotely conducted Early Treatment Study vs those in the clinic-based study) to improve accuracy of comparison given that the PEP Trial enrolled participants with COVID-19 exposures and thus had different enrollment criteria. All analyses and mapping were done in R statistical software version 4.0 (R Project for Statistical Computing). Data were analyzed from April to August 2021.

## Results

A total of 1410 participants were included. The remote trials enrolled a total of 1160 participants (231 participants in the Early Treatment Study and 929 participants in the Hydroxychloroquine COVID-19 Postexposure Prophylaxis Study) from 41 states. The mean (range) age of remote trial participants was 39 (18-80) years, and 676 individuals (58.3%) reported female sex as assigned at birth. Of 250 participants enrolled in the clinic-based study, the mean (range) age was 50 (19-79) years and 131 individuals (52.4%) reported female sex as assigned at birth. Participants in remote trials collected and shipped 14 380 of 15 890 expected nasal swabs (90.5%) for the primary outcome. Retention was high for remote trials, with participants completing 17 129 of 18 893 expected surveys (90.7%) over the follow-up period.

Racial, ethnic, and geographic diversity were observed at increased proportions in the remotely conducted Early Treatment Study (Table 1).^10,11,12^ Among 228 participants in the Early Treatment Study with race data vs participants in the clinic-based Expanded Access to Convalescent Plasma for the Treatment of Patients With COVID-19 study, 39 individuals (17.1%) vs 1 individual (0.4%) identified as Alaska Native or American Indian, 11 individuals (4.8%) vs 22 individuals (8.8%) identified as Asian, 26 individuals (11.4%) vs 4 individuals (1.6%) identified as Black, 3 individuals (1.3%) vs 1 individual identified as Native Hawaiian or Pacific Islander, 117 individuals (51.3%) vs 214 individuals (85.6%) identified as White, and 32 individuals (14.0%) vs 8 individuals (3.2%) identified as other race (*P* < .001). Among 230 individuals in the Early Treatment Study vs 236 individuals in the clinic-based trial with ethnicity data, 71 individuals (30.9%) vs 11 individuals (4.7%) identified as Hispanic or Latinx (*P* < .001). There were 29 individuals in the Early Treatment Study with nonurban residences (ie, rural, small town, or peri-urban; 12.6%) vs 6 of 248 individuals in the clinic-based trial with residence data (2.4%) (*P* < .001). When restricted to the same catchment area (250 miles from Seattle, Washington), 40 of 127 individuals in the Early Treatment Study were White and non-Hispanic (31.5%) compared with 202 of 248 individuals in the clinic-based study (81.5%) (*P* < .001).

**Table 1.** Demographic Data for Remote and Clinic-Based Studies

Characteristic	Participants with COVID-19, No. (%)^a^			*P* value for Early Treatment Study vs clinic-based study^b^^,^^c^
	Remotely conducted studies		Clinic-based study: Expanded Access to Convalescent Plasma for the Treatment of Patients With COVID-19 study (N = 250)^12^	
	Hydroxychloroquine COVID-19 PEP Study (N = 929)^11^	Early Treatment Study (N = 231)^10^		
Age, mean (SD), y	39 (15)	39 (13)	50 (14)	<.001
Sex (assigned at birth)				
Female	545 (58.7)	131 (56.7)	131 (52.4)	.36
Male	382 (41.1)	100 (43.3)	119 (47.6)	
Other	2 (0.2)	0	0	
Racial identity, No./No. (%)				
Alaska Native or American Indian	18/913 (2.0)	39/228 (17.1)	1 (0.4)	<.001
Asian	87/913 (9.5)	11/228 (4.8)	22 (8.8)	
Black or African American	88/913 (9.6)	26/228 (11.4)	4 (1.6)	
Native Hawaiian or Pacific Islander	6/913 (0.7)	3/228 (1.3)	1 (0.4)	
White	599/913 (65.6)	117/228 (51.3)	214 (85.6)	
Other^d^	115/913 (12.6)	32/228 (14.0)	8 (3.2)	
Hispanic or Latinx identifying, No./No. (%)	222/928 (23.9)	71/230 (30.9)	11/236 (4.7)	<.001
Primary language				
English	852 (91.7)	210 (90.9)	250 (100)	<.001
Spanish	77 (8.3)	21 (9.1)	0	
Community zip code				
Rural	4 (0.4)	1 (0.4)	2/248 (0.8)	<.001
Small town	9 (1.0)	2 (0.9)	1/248 (0.4)	
Peri-urban	39 (4.2)	26 (11.3)	3/248 (1.2)	
Urban	877 (94.4)	202 (87.4)	242/248 (97.6)	

Abbreviation: PEP, Postexposure Prophylaxis.

^a^ Denominators provided only when missing data.

^b^ Post hoc comparison is presented for participants with COVID-19 infection enrolled in the remotely conducted Early Treatment Study and clinic-based Expanded Access to Convalescent Plasma for the Treatment of Patients With COVID-19 study. This was done to improve accuracy of comparison given that the Hydroxychloroquine COVID-19 PEP Trial enrolled participants with COVID-19 exposures and thus had different enrollment criteria.

^c^ *P* values are from Fisher exact tests, except for age, which is from a *t* test.

^d^ Self-reported racial identity categories included the option other if racial identity was not captured in listed categories.

A total of 307 632 unique users clicked on online recruitment advertisements for the remote trials, and 125 147 (40.7%) unique users had age demographics associated with their Google Analytics data. Of these, more than half (84 188 individuals [67.3%]) engaged via Facebook compared with 35 029 individuals (28.0%) via Google, 1753 individuals (1.4%) via Twitter, and 4255 individuals (3.4%) via other, smaller platforms. There were 10 455 online users who clicked through the advertisement and accessed the prescreening survey (7.5%), with the highest rate among users ages 18 to 24 years on Facebook (605 of 6232 individuals [9.7%]) (Table 2). Clinician referrals to the study website and linked referrals from personal social media posts were rarely observed.

**Table 2.** Online Engagement With Targeted Advertising by Age Range^a^

Age group	Unique users, No. (%)	Users accessed prescreening (goal conversion rate), No. (%)^b^	Facebook ad		Google ad	
			Unique users, No.	Users accessed prescreening (goal conversion rate), No. (%)	Unique users, No.	Users accessed prescreening (goal conversion rate), No. (%)
All ages	125 147	10 455 (8.4)	84 188	7385 (8.8%)	35 029	1032 (2.9%).
Age range, y						
18-24	9637 (7.7)	913 (9.5)	6232	605 (9.7)	2603	84 (3.2)
25-34	26 249 (21.0)	2254 (8.6)	17 996	1602 (8.9)	6610	179 (2.7)
35-44	25 201 (20.1)	2233 (8.9)	17 999	1656 (9.2)	6191	199 (3.2)
45-54	26 924 (21.5)	2442 (9.1)	19 542	1798 (9.2)	6493	215 (3.3)
55-64	22 190 (17.7)	1643 (7.4)	14 318	1132 (7.9)	6990	189 (2.7)
≥65	14 946 (11.9)	970 (6.5)	8101	592 (7.3)	6142	166 (2.7)

^a^ Online users without age associated with Google Analytics were excluded.

^b^ The goal conversion rate refers to the frequency of online users who clicked through the advertisement and accessed the prescreening survey (No. of users accessing prescreening divided by number of unique users).

Geographic distribution was centered on trial sites. Participants were enrolled a median (IQR; maximum) 19 (7-129; 4961) miles from each trial site in the remote trials and 11 (7-19; 208) miles from the clinic-based study site (Figure). Enrollment of peri-urban–dwelling participants was increased in the remote trials; however, enrollment of rural-dwelling participants was rare in both types of trials (Table 1).

**Figure.**
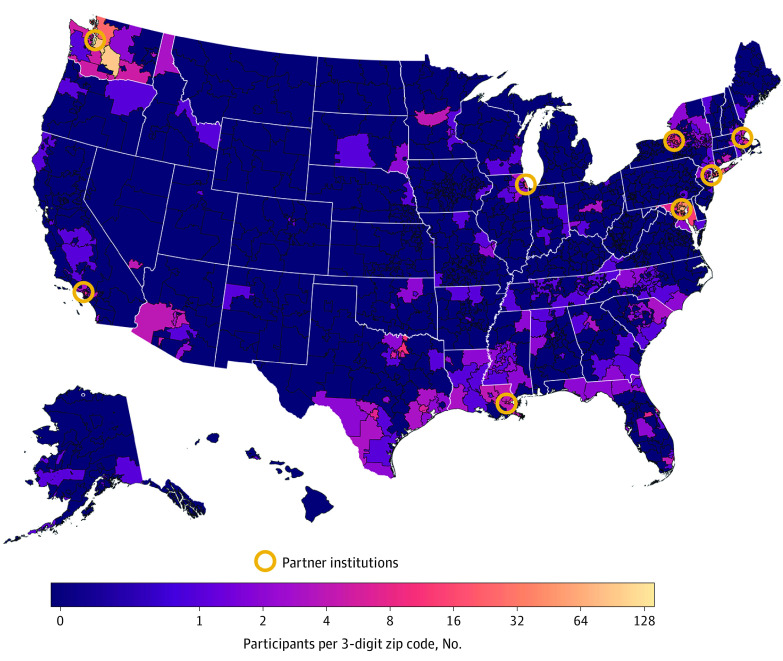
Heat Map of Participant Location vs Remote Study Site Institutions Location is given by 3-digit ZIP Code Tabulation Area vs remote study site institution. Circles indicate sites of partner institutions of remote studies.

## Discussion

In this cohort study, racial, ethnic, and geographic diversity were observed at increased proportions in the remotely conducted Early Treatment Study. These findings suggest that remote COVID-19 trial participation may be an important step toward inclusive evidence for public health advancement. In remote trials, protocols may be conducted with high fidelity and safety monitoring,^10,14^ without risking the spread of SARS-CoV-2 or other respiratory viruses.

Completely remote trial designs with inclusive social media strategies may address some of the structural barriers that disproportionally impact members of racial and ethnic minority groups in the US. Further work is needed to make clinical research more inclusive and address structural racism and the urban-rural divide that impact access to clinical trials.^5,7,8^ While remote trials in this study enrolled a higher proportion of participants outside of large urban centers than the in-clinic comparison study, additional research regarding geographic inclusion is needed given that the proportion of rural participants was low.

### Limitations

There are several limitations to this analysis. Online analytic data does not capture the racial or ethnic identity of users. Comparison between completely remote trials and our clinic-based study was post hoc, and disease severity and motivations for participation may differ significantly among participants of a treatment trial, prevention trial, and postillness study. To minimize these differences, statistical comparison was limited by confirmed COVID-19 infections to the remotely conducted Early Treatment Study compared with the clinic-based study. While we found that some aspects of diversity were increased among participants in remote vs clinic-based studies, we did not examine other elements of demographic diversity (eg, socioeconomic status or health literacy) and did not evaluate whether trial participation was representative of community COVID-19 prevalence. We did not capture indirect benefits associated with a remotely conducted trial. These include the shift to fragmented time commitment, because participants must self-collect specimens and respond to surveys, rather than time needed to travel to and attend appointments, which may be associated with reduced barriers to participation for participants with childcare needs and fixed working hours. We did not collect data on differences among study personnel characteristics, technical skills, or expertise or study operational costs. Future work is needed to investigate whether these findings may extend beyond the context of the COVID-19 pandemic.

## Conclusions

This study found that demographic and geographic diversity were increased in the remotely conducted Early Treatment Study vs a clinic-based trial. Our findings suggest that remote trial participation and online recruitment may be associated with improved research equity and inclusion of participants from more diverse racial, ethnic, and geographic communities.
