# Errors in Byline, Author Affiliations, and Author Contributions

**DOI:** 10.1001/jamanetworkopen.2022.3146

**Published:** 2022-03-09

In the Original Investigation titled “Comparison of Racial, Ethnic, and Geographic Location Diversity of Participants Enrolled in Clinic-Based vs 2 Remote COVID-19 Clinical Trials,”^1^ published February 14, 2022, there were spelling errors in the names of Dr Barnabas in the byline and Dr Boonyaratanakornkit in the byline and Author Affiliations and Author Contributions sections at the end of the article. This article has been corrected.^1^

## References

[1] Stewart J, Krows ML, Schaafsma TT, . Comparison of racial, ethnic, and geographic location diversity of participants enrolled in clinic-based vs 2 remote COVID-19 clinical trials. JAMA Netw Open. 2022;5(2):e2148325. doi:10.1001/jamanetworkopen.2021.48325 35157053 PMC8844998

